# Longitudinal impact of parent-teacher relationship on middle school students’ academic achievements in China

**DOI:** 10.3389/fpsyg.2022.872301

**Published:** 2022-08-23

**Authors:** Wangqian Fu, Qianqian Pan, Ying Yuan, Guanyu Chen

**Affiliations:** ^1^Faculty of Education, Beijing Normal University, Beijing, China; ^2^Centre for Research in Pedagogy and Practice, Office of Education Research, National Institute of Education, Nanyang Technological University, Singapore, Singapore; ^3^School of Journalism and Communication, Beijing Normal University, Beijing, China; ^4^Department of Educational and Counselling Psychology and Special Education, Faculty of Education, The University of British Columbia, Vancouver, BC, Canada

**Keywords:** parent-teacher relationship, middle school, academic achievements, mainland China, hierarchical linear model

## Abstract

**Objective:**

The study aims to discuss the longitudinal impact of the parent-teacher relationship on students’ academic achievements in China.

**Method:**

Based on the China Education Panel Survey, covering the data from 438 classes of 112 schools in 28 county-level administrative areas in China, we used the hierarchical linear model to analyze the data.

**Results:**

We found that the parents’ active communication with teachers, parents’ participation in parent meetings, teachers’ active contact, whether parents are afraid to communicate with teachers, and parents’ willingness to participate in parent meetings have significant relationships with students’ academic achievements. At the class level, the extent of teachers’ stress from parents’ requests and teachers’ perception of respect from parents also affected students’ academic achievements significantly in the Chinese context.

**Conclusion:**

There was a longitudinal association between the parent-teacher relationship and students’ academic achievements. The practical implication was discussed in the paper.

## Introduction

Family and school are two important environments closely related to adolescents, and cooperation between the two greatly impacts adolescents’ development (Lohman et al., 2007; Guo and Zhang, 2015). The Chinese government realizes the importance of home-school cooperation and has recently promulgated a series of policy documents to promote home-school cooperation. In 2017, the “School Management Standards for Compulsory Education” proposed that harmonious relationships among family, school, and community should be built to enhance parents’ participation in school governance and form a joint effort to educate people (Ministry of Education, 2017). Although the government is attaching increasing importance to home-school cooperation, there are still many problems with parent-teacher relationships in China. For example, the status of parents and teachers is unequal, showing the characteristics of a one-way communication model, a single form of cooperation under which parents lack the initiative to speak and where the communication channels are not smooth between parents and teachers (Zhang, 2011; Yang and Liu, 2012). Since parents and teachers are the two crucial participants in a child’s education, their interactions heavily influence child outcomes (Serpell and Mashburn, 2012). Especially the parent-teacher relationship is critical to the children’s social-emotional development (Lang et al., 2020) and their academic outcome (Kim and Hill, 2015). Despite existing research having paid attention to the association between parent-teacher relationships and students’ academic outcomes, little is known about the long-term effects of parent-teacher relationships on students’ outcomes; especially in China, most classroom teachers teach the same class of students from the enrollment to graduation in middle schools. Therefore, it is essential to investigate whether the impacts of parent-teacher relationships could be carried over. Together, the current study aims to discuss the longitudinal impact of the parent-teacher relationship on students’ academic achievements in China.

### The influence of parent-teacher relationships on students’ development

Dozens of studies show that good family school relationships can promote students’ academic achievements and positively impact students’ growth (Garcia, 2004; Pomerantz et al., 2007; Zhang, 2011; Kim and Hill, 2015; Herman and Reinke, 2017). According to the Ecological systems theory (Bronfenbrenner, 1979; Bronfenbrenner and Morris, 1998), adolescent development is influenced directly by the interactions that take place within a single microsystem (such as the family, school, or peer group), as well as the similarities and differences in the pattern of the interactions that occur across these systems. Family and school are the two most important environments closely associated with adolescent development (Lohman et al., 2007; Santana et al., 2016).

Research shows that not all family school relationships are good for students (Pomerantz et al., 2007), and it depends on whether parents and teachers have good relations (Nir and Ami, 2005; Emerson et al., 2012; Liang, 2015). Only a relationship based on mutual trust and honesty can rich and frequent interaction be conducive to the development of students (Constantino, 2003; Garcia, 2004). A good relationship between parents and teachers can provide consistent support for students, making students more likely to recognize the benefits of learning (Harris and Goodall, 2009; Emerson et al., 2012). Waller (1932) points out that parents and teachers are natural enemies, not natural collaborators. The complex and crucial connection between families and schools is embodied in the relationships between individual teachers and their students’ families (Driessen et al., 2005; Cole, 2013).

### The connotation of the parent-teacher relationship

Good parent-teacher relationships are generally effective at maximizing students’ potential, based on the mutually rich and frequent interactions built upon trust and honesty (Constantino, 2003; Garcia, 2004). According to interpersonal communication theory, the interpersonal relationship measurement can be classified into two variables. The first is the dimension of parent-teacher contact, which considers the individual’s judgment of the relationship, such as the frequency of communication, the content, and means of communication, and the efforts the other may make to maintain the relationship. The second is the parent-teacher relationship quality, which mainly refers to the intensity of this judgment and is the perception of the intensity of the former (Dillard et al., 1999). Generally, an individual’s perception of the quality of a relationship comes directly from the state of the communication, such as the frequency of communication and the degree of self-disclosure. Relationships develop over time through a process of self-disclosure (Bylund et al., 2012). Moreover, a relationship comes from the perception of the individual’s influence on others, such as how much influence an individual can have on the decisions or behaviors of others and their evaluation of others as individuals (Zhang, 2016). Such that, there is a difference between parent-teacher contact and the parent-teacher relationship quality. Parent-teacher contact refers to the frequency of interactions between parents and teachers, whereas the parent-teacher relationship quality refers to the quality of their relationship as well as how well their goals are aligned (Serpell and Mashburn, 2012; Stormont et al., 2013; Deng et al., 2018).

In parent-teacher contact, the frequency of ties and activities between families and schools influences the relationship between parents and teachers (Zhao and Hong, 2012). The more connections and activities between families and schools are built and held, the better the relationship between teachers and parents will be (Adams and Christenson, 2000; Minke, 2006). The more frequent the interaction between parents and teachers, the more likely parents are to pass on their educational expectations to their children, thus promoting students’ improvement at the cognitive level and success in academic achievement (Goyette and Xie, 1999; Sebastian and Allensworth, 2012). Parents’ participation in parental meetings, voluntary school activities, and initiatives where they learn from teachers about their children’s learning or behavior can significantly improve their student’s access to school (Perna and Titus, 2005; Pong et al., 2005). Regular parental meetings encourage parents to spend more time helping students and supervising school work, which is beneficial to improving students’ test scores and plays an important role in student attitudes and behaviors (Islam, 2019). Together, parents’ participation in parental meetings, school activities, and teacher contact has a significant positive impact on students with low academic achievements. Furthermore, parental behavior can help students understand the importance that their parents attach to school as well as to their learning behavior (Cheng and Li, 2015; Li, 2017).

In addition, parent-teacher relationship quality variables means the perception of self-influence on others, such as the extent to which one influences other people’s decisions, actions, or evaluations (Zhang, 2016). The subjective relationship quality in the parent-teacher relationship refers to the parents’ and teachers’ emotional perception of the parent-teacher interaction. On the one hand, if parents do not experience a welcoming environment or support from teachers, their participation in constructing the parent-teacher relationship will be reduced. On the other hand, whether teachers receive due respect has a connection to the teachers’ work enthusiasm and students’ academic performance (Chen, 2008). If parents exhibit negative emotions toward the parent-teacher relationship, it will be harder to construct an excellent parent-teacher relationship (Tao, 2016). So, the emotional experience has an important impact on the parent-teacher relationship and students’ academic performance.

### The parent-teacher relationship in mainland China

Previous studies have shown that contact and communication between teachers and parents are not frequent enough in China (Li, 2013; Zhu and Gao, 2014; Zhang, 2018). It is also possible that schools in China are usually closed, and school-based communication is dominated by schools (Ma, 2010; Li, 2013). Therefore, parents’ participation in school education is limited. At present, the parent-teacher relationship in China is usually manifested by a teacher telling parents about their children’s behavior in school, and then the parents participate in their children’s school education accordingly (Li, 2013; Huang, 2016). From there, most of the links between teachers and parents occur when a student exhibits bad performance, and parents naturally hope that the fewer links there are, the better everyone will be (Zhang, 2003). On the other hand, teachers report that their relationships with parents are a major source of occupational stress (Grolnick and Seal, 2008; Pepe and Addimando, 2013, 2014). Teachers feel more unpleasant experiences are involved in dealing with parent relationships than in other aspects of teaching (Hargreaves and Fullan, 1998).

Although many research studies have shown that the parent-teacher relationship has an important impact on student’s academic achievements (Hill and Tyson, 2009), the parent-teacher relationship in home-school cooperation has escaped the attention of many Chinese researchers, especially in regard to the lack of quantitative research. There are some limitations on the previous study on the topic. Firstly, the existing studies focus on the status of the parent-teacher relationship and the association between the parent-teacher relationship and students’ academic outcome at a certain period (Yuan et al., 2019), that is the use of cross-sectional data. And the long-term effect of the parent-teacher relationship on academic achievement is rare. The unique current social culture and educational environment of mainland China may limit the generalizations of dominant Western research findings on the parent-teacher partnership in China (Deng et al., 2018). Especially the teachers in a public school in China will widely be in charge of the same classes in the middle school; that is, the same teachers teach students in the 3 years of middle school in China, which may lead to the parent-teacher relationship having a carry-over impact. Since teachers will teach the same class for a long time, the original parent-teacher relationship may give parents and teachers a fixed impression, which will have a long-term impact on their interaction, and then have a long-term impact on students’ performance (Deng et al., 2018). Secondly, previous studies take the parent-teacher relationship as a whole variable while didn’t discuss the influence of the parent-teacher relationship at a different level. Assessing different aspects of the parent-teacher relationship may provide more detailed evidence to improve the parent-teacher relationship. Therefore, this paper attempts to address the gaps by aiming to examine the longitudinal association between the parent-teacher relationship and students’ academic achievements in China.

Adolescent development involves various factors, including the social, emotional, and cognitive aspects of the transition from childhood to adulthood (Jaggers et al., 2015). Compared with the performance at the primary school stage, middle school students’ academic performance tends to decline because of the great changes in puberty and their environment (Steinberg and Silk, 2002; Barber and Olsen, 2004; Keating, 2004; Lerner and Steinberg, 2004; Smetana et al., 2004; Hill and Chao, 2009). Additionally, the independence of middle school students in all aspects has gradually increased, resulting in a less close parent-teacher relationship than the one that exists in kindergartens and primary schools, and effective cooperation may be reduced (Seginer, 2006). Therefore, it is worthwhile to use middle school students as research subjects to explore the association between the parent-teacher relationship and middle school students’ developmental outcomes in the context of mainland China as well as the evidence-based potential practical strategies.

This study employs the hierarchical linear model (HLM) to discuss the association between the parent-teacher relationship and middle school students’ academic achievements by analyzing China Education Panel Survey (CEPS) data, which is a large-scale, nationally representative, longitudinal public database. Two questions were addressed in this study to enhance our understanding of the parent-teacher relationship in mainland China:

(1) Does the parent-teacher relationship have a carry-over impact on middle school students’ academic achievements at the parent-child dyad level^1^ ?
(2) Does the parent-teacher relationship have a carry-over impact on middle school students academic achievements at the class level?

## Methodology

### Data source

The data used in this study are from a large-scale longitudinal study [2013–2014 school year (Wave 1)] and [2014–2015 school year (Wave 2)] of the CEPS, a project conducted by the National Survey Research Center (NSRC) at the Renmin University of China. The respondents were middle school students in seventh grade and ninth grade. The sampling method was stratified random sampling based on the average education level and the proportion of migrants in the whole population. In addition, the respondents included parents, teachers, and school administrators. The survey covered 438 classes from 112 schools in 28 county-level administrative areas throughout the country; due to the compulsory education law (Ministry of Education, 2021) in China, the government administrates compulsory education mainly at the county level. The CEPS survey can represent the overall education situation in China. The questionnaire survey procedure starts with the investigator of NSRC contacting the principals at the selected middle schools and then collecting the students’ data by questionnaire.

The students’ ability scores were compiled according to the national students’ cognitive ability on a 20-item test designed by NSRC, which includes the ability of language, graph, calculation, and logic. The score ranges from 0 to 20. It was completed on the spot in class and was strictly time-controlled. The answers were collected and sealed on the spot. The principals then arranged for the related teachers to fill out the questionnaire to collect the teachers’ data. After that, the head teachers of the selected classes asked the parents to fill out the questionnaire. Finally, the questionnaire was mailed to NSRC after it was collected by the head teachers. Many studies have analyzed and discussed the problems of education in China based on these data (e.g., Huang, 2016; Wu, 2016; Zhang, 2016; Li and Zheng, 2017; Pan and Zhang, 2017; Tong, 2017; Wei and Ma, 2017; Zhao et al., 2017; Fang, 2018; Liang et al., 2018). The data are very rich, including basic personal information, students’ situations in school, parent-child interactions, and home-school interactions, all of which can provide information for us to further study the impacts of the parent-teacher relationship on students’ academic achievements.

In this study, we used the data of students in Grade 7 to analyze the carry-over impact of the parent-teacher relationship on students’ achievements for middle school students in China, since the students in seventh grade have the follow-up data 1 year later. After excluding incomplete samples, the sample size of students (parents) was 8,426, and the sample size of classes was 185.

### Variables

Data used in the current study were collected from three sources, including a child’s responses, responses from one of his/her parents, and responses from his/her classroom teachers. Therefore, we classified variables into two levels, parent-child dyad level and class level.

#### Student variables

Students’ academic achievement of 2014–2015 (wave-2) was the dependent variable and cognitive ability simultaneously served as a control variable.

Regarding Students’ academic achievement (SAA), CEPS collected students’ original mid-term Chinese, math, and English scores in the autumn semester of 2014, which schools provided. Since the full raw scores of each subject were different across schools, we computed students’ academic scores *via* dividing their gained raw scores by the full raw scores, indicating the percentage of correct of each subject, and then the averaged percentage of correct of three subjects was computed as a measure of students’ SAA. The students’ cognitive ability scores were compiled according to the national students’ cognitive ability on a 20 items test designed by NSRC, which includes the ability of language, graph, calculation, and logic. The score ranges from 0 to 20. Also, Students’ gender was included as a control variable.

#### Parent-teacher relationship variables

This study measured the parent-teacher relationship on two levels: child-level and class-level. They were all collected in the 2013–2014 school year (wave-1). At the parent-child dyad level, parents reported in five following questions:

(1) Parents’ participation. Do this child’s parents attend the parent meeting this semester? There were four options, including (a)The school held one, and the parents did attend; (b) The school held one, but the parents didn’t attend; (c) Not held yet, but the parents are going to attend once held; and (d)Not held yet, and the parents are not going to attend if held. We recoded (a) and (c) as 1 to represent parents’ active attitude on it. (See PP in Table 1).

**TABLE 1:** Research variables.

Levels	Variables		Codes
Level 1: Parent-child dyad level Responses from Child and Parents	Wave 2 student academic achievement	W2-SAA	Average scores across Chinese, Math, and English
	Wave 2 Cognitive ability	W2-CA	Standardized total score of the student’s cognitive ability test estimated by the three parameter IRT model.
	Student gender	SG	0 = female, 1 = male
	Parental education level	PEL	1 = no education, 2 = primary school, 3 = secondary school, 4 = technical secondary school, 5 = vocational high school, 6 = high school, 6 = associate degree, 7 = bachelor, 8 = master and above
	Family economic status	FES-C1	Difficult reference group
		FES-C2	Medium
		FES-C3	Rich
	Wave 1 parents’ participation/willing to participate in parents’ meeting	PP	0 = non-participation, 1 = participation
	Wave 1 frequency of parents contact with teachers actively in this semester	PCTA	1 = none, 2 = once, 3 = two to four times, 4 = five times and above
	Wave 1 frequency of teachers contact with parents actively in this semester	TCPA	1 = none, 2 = once, 3 = two to four times, 4 = five times and above
	Wave 1 extent of parents’ afraid of communicating with teachers	PA	1 = quite afraid, 2 = a little bit afraid, 3 = not afraid at all
	Wave 1 parents cooperate with the teacher’s requirement	PCTR	1 = complete, 2 = mostly, 3 = rarely, 4 = not at all
Level 2: class-level Responses from teachers	Wave 1 teachers gender	TG	0 = female, 1 = male
	Wave 1 numbers of parents that teachers know	NPTK	1 = all most none, 2 = a few, 3 = half of a class, 4 = most of a class, 5 = every parent of a class
	Wave 1 extent if teachers’ stress from parents’ request	TSPR	1 = not at all, 2 = to a small extent, 3 = to a general extent, 4 = to a large extent, 5 = to an extremely large extent
	Wave 1 teachers’ perception of respect from parents	TPRP	1 = none, 2 = just a few, 3 = about a half, 4 = more than half, 5 = all of them
	Wave 1 teaching experiences	TA	Mean = 15, SD = 8.97, Median = 14

Note. Wave 1 = 2013–2014 school year, Wave 2 = 2014–2015 school year.

(2) Frequency of parents contact with teachers actively. How many times have this child’s parents contacted the teacher at school this semester? Parents responded it on a 4-point scale (1 = Never, 2 = Once, 3 = Two to four times, and 4 = Five times or more). (See PCTA in Table 1).
(3) Frequency of teachers contact with parents actively. How many times has this child’s teacher contacted the parents this semester? Parents responded it on a 4-point scale (1 = Never, 2 = Once, 3 = Two to four times, and 4 = Five times or more). (See TCTA in Table 1).
(4) Parents’ afraid. Are you afraid of communicating with the school teacher? Parents reported it on a 3-point scale (1 = Quite afraid, 2 = A little bit afraid, and 3 = Not afraid at all). (See PA in Table 1).
(5) Parents cooperate with the teacher’s requirement. To what extent could you meet the requirement this semester if the teachers require the parents to check their child’s homework, such as writing “checked,” signing names, etc.? Parents responded it on a 4-point scale (1 = Complete, 2 = Mostly, 3 = Rarely, and 4 = Not at all). (See PCTR in Table 1).

At the class level, the classroom teacher responded to three questions to represent the parent-teacher relationship of a particular classroom.

(1) Numbers of parents that teachers know. How many parents of your students do you know about? Teachers responded it on a 5-point scale (1 = None, 2 = Just a few, 3 = About a half, 4 = More than half, and 5 = All of them). (See NPTK in Table 1).
(2) Teachers’ stress from parents’ request. How much pressure do you have concerning the requirements of students’ parents. Teachers responded it on a 5-point scale (1 = None, 2 = Low, 3 = Moderate, 4 = High, and 5 = Very high). (See TSRP in Table 1).
(3) Teachers’ perception of respect from parents. Generally speaking, how many parents of your students pay respect to you? Teachers responded it on a 5-point scale (1 = None, 2 = Just a few, 3 = About a half, 4 = More than half, and 5 = All of them). (See TPRP in Table 1).

In line with existing research, the measures of the parent-teacher relationship also reflected both the parent-teacher contact and parent-teacher relationship quality. More specifically, parents’ active communication with teachers, teachers’ active contact with parents, parents’ participation in parental meetings, the number of parents that the teacher knows, and how parents cooperate with the teacher’s requirements represented the parent-teacher contact. Meanwhile, whether parents are afraid to communicate with teachers, teachers’ stress from parents’ requests and teachers’ perception of respect from parents reflect parent-teacher relationship quality.

Besides the aforementioned variables, we also included parental education level, family economic status (FES), teachers’ gender, and teachers’ teaching experience (The cumulative time for teachers to engage in teaching work which is measured by year) as control variables. To be noted, we merged the FES into three categories (Difficulty, Medium, and Rich) from a five-category variable (very difficult, some difficult, medium, rich, and very rich) from the original database by merging the first two categories and last two categories due to the small numbers in the first and last categories. (See Table 1 for details).

### Analysis method

Due to the data hierarchy, where students were nested in the classroom, the HLM was adopted to account for the dependency among students in the same classroom. Considering that there are only two classes in each grade in each school, it is not suitable to construct a three-level model of student, class, and school. This paper uses a two-level HLM as the analysis model. Level 1 is the parent-child dyad level, and students’ gender, cognitive abilities, parents reported parent-teacher relationship variables, FES used as the predictors. Considering that categorized variables cannot be directly used in the HLM, a dummy coding method was used to transform the FES into two dummy variables. More specially, the first categorical of FES (FES-C1, difficulty level) was set as the reference level, and then two dummy variables, FES.C2 and FES.C3, represented medium and rich levels, respectively, were created.

Level 2 is the class level, and the teachers reported that parent-teacher variables were used as class-level predictors. In addition, corresponding child-level predictors were aggregated at the class level to separate level-1 and level-2 effects (Hoffman, 2015, pp. 344).

### Model

We followed the step-up model-building approach as suggested by Raudenbush and Bryk (2002) in the current study.

Step 1: a null model with no predictors were tested to estimate the interclass correlation of the outcome variables.

Model 0:


(1) $$\text{Level}-1\,\mathrm{Model}:Y_{\text{ij}}=\beta_{0\,j}+r_{\text{ij}}$$

(2) $$\text{Level}-2\,\mathrm{Model}:\beta_{0\,j}=\gamma_{00}+u_{0\,j}$$

In this model, *j* represents different classes; *i* stands for different students; *Y*_*ij*_ corresponds to the academic achievement of student *i* in class *j*; β_*0j*_ is the mean of the students’ academic achievements in class *j*; *r*_*ij*_ is the difference between student *i* and the class mean; Var(*r*_ij_) reflects the random error at the student/parent level; γ_*00*_ is the mean of all students’ academic achievements; *u*_*0j*_ is the difference between the class mean and the all-student mean; andVar(*u*_0*j*_) reflects the error of the class level.

Step 2: a random intercept model was tested with all level-1 predictors centered at the class mean and corresponding level-2 predictors by aggregating at the class-level to separate level-1 and level-2 effects (Hoffman, 2015, pp. 344).


(3) $$\text{Level}-1\,\mathrm{Model}:Y_{\text{ij}}=\beta_{0\,j}+\beta_{1\,j}\,{\left(\text{SG}\right)}_{\text{ij}}+\beta_{2\,j}\,{\left(\text{W}\,2\,\text{CA}\right)}_{\text{ij}}+\beta_{3\,j}\,{\left(\text{PEL}\right)}_{\text{ij}}+\beta_{4\,j}{\left(\mathrm{FES}.\mathrm{C2}\right)}_{\text{ij}}+\beta_{5\,j}{\left(\mathrm{FES}.\mathrm{C3}\right)}_{\text{ij}}+\beta_{6\,j}{\left(\text{PP}\right)}_{\text{ij}}+\beta_{7\,j}{\left(\text{PCTA}\right)}_{\text{ij}}+\beta_{8\,j}\,{\left(\text{TCPA}\right)}_{\text{ij}}+\beta_{9\,j}\,{\left(\text{PA}\right)}_{\text{ij}}+\beta_{10\,j}\,{\left(\text{PCTR}\right)}_{\text{ij}}+r_{\text{ij}}$$

(4) $$\text{Level}-2\,\mathrm{Model}:\beta_{0\,j}=\gamma_{00}+\gamma_{01}\,{\left(\text{SGCM}\right)}_{j}+\gamma_{02}\,{\left(\text{W}\,2\,\text{CACM}\right)}_{j}+\gamma_{03}\,{\left(\text{PELCM}\right)}_{j}+\gamma_{04}{\left(\mathrm{FES}.\mathrm{c2CM}\right)}_{j}+\gamma_{05}{\left(\mathrm{FES}.\mathrm{c3CM}\right)}_{j}+\gamma_{06}{\left(\text{PPCM}\right)}_{j}+\gamma_{07}\,{\left(\text{PCTACM}\right)}_{j}+\gamma_{08}\,{\left(\text{TCPACM}\right)}_{j}+\gamma_{09}\,{\left(\text{PACM}\right)}_{j}+\gamma_{010}\,{\left(\text{PCTRCM}\right)}_{j}+u_{0\,j}$$

(5) $$\beta_{k\,j}=\gamma_{1\,k}$$

Where, *k* represents the *k^th^* level-1 predictors, *k* ∈ [1,10], meaning no random slopes were specified in this step.

Since SG was a categorical variable and the female as the reference group, the percentage of male students in each classroom was calculated as the level-2 predictor (SGCM). Similarly, the percentage of FES.C2 and FES.C3 at each classroom were computed as the level-2 predictors.

Step 3: a series of random slope models were tested to examine which level-1 coefficients varied significantly across classrooms *via* conducting a likelihood ratio test to compare models with and without the random slopes.


(6) $$\text{Level}-1\,\mathrm{Model}:Y_{\text{ij}}=\beta_{0\,j}+\beta_{1\,j}\,{\left(\text{SG}\right)}_{\text{ij}}+\beta_{2\,j}\,{\left(\text{W}\,2\,\text{CA}\right)}_{\text{ij}}+\beta_{3\,j}\,{\left(\text{PEL}\right)}_{\text{ij}}+\beta_{4\,j}{\left(\mathrm{FES}.\mathrm{C2}\right)}_{\text{ij}}+\beta_{5\,j}{\left(\mathrm{FES}.\mathrm{C3}\right)}_{\text{ij}}+\beta_{6\,j}{\left(\text{PP}\right)}_{\text{ij}}+\beta_{7\,j}{\left(\text{PCTA}\right)}_{\text{ij}}+\beta_{8\,j}\,{\left(\text{TCPA}\right)}_{\text{ij}}+\beta_{9\,j}\,{\left(\text{PA}\right)}_{\text{ij}}+\beta_{10\,j}\,{\left(\text{PCTR}\right)}_{\text{ij}}+r_{\text{ij}}$$

(7) $$\text{Level}-2\,\mathrm{Model}:\beta_{0\,j}=\gamma_{00}+\gamma_{01}\,{\left(\text{SGCM}\right)}_{j}+\gamma_{02}\,{\left(\text{W}\,2\,\text{CACM}\right)}_{j}+\gamma_{03}\,{\left(\text{PELCM}\right)}_{j}+\gamma_{04}{\left(\mathrm{FES}.\mathrm{c2CM}\right)}_{j}+\gamma_{05}{\left(\mathrm{FES}.\mathrm{c3CM}\right)}_{j}+\gamma_{06}{\left(\text{PPCM}\right)}_{j}+\gamma_{07}\,{\left(\text{PCTACM}\right)}_{j}+\gamma_{08}\,{\left(\text{TCPACM}\right)}_{j}+\gamma_{09}\,{\left(\text{PACM}\right)}_{j}+\gamma_{010}\,{\left(\text{PCTRCM}\right)}_{j}+u_{0\,j}$$

(8) $$\beta_{\text{kj}}=\gamma_{10}+\mu_{\text{kj}}$$

Where, *k* represents the *k*^th^ level-1 predictors, *k* ∈ [1,10],μ_*kj*_ indicates random slopes were specified in this step. Only the significant random slopes were kept to keep the model as parsimony as possible.

Step 4: class-level predictors were added to models to examine if these class-level predictors could explain the variance of random intercept.


(9) $$\text{Level}-1\,\mathrm{Model}:Y_{\text{ij}}=\beta_{0\,j}+\beta_{1\,j}\,{\left(\text{SG}\right)}_{\text{ij}}+\beta_{2\,j}\,{\left(\text{W}\,2\,\text{CA}\right)}_{\text{ij}}+\beta_{3\,j}\,{\left(\text{PEL}\right)}_{\text{ij}}+\beta_{4\,j}{\left(\mathrm{FES}.\mathrm{C2}\right)}_{\text{ij}}+\beta_{5\,j}{\left(\mathrm{FES}.\mathrm{C3}\right)}_{\text{ij}}+\beta_{6\,j}{\left(\text{PP}\right)}_{\text{ij}}+\beta_{7\,j}{\left(\text{PCTA}\right)}_{\text{ij}}+\beta_{8\,j}\,{\left(\text{TCPA}\right)}_{\text{ij}}+\beta_{9\,j}\,{\left(\text{PA}\right)}_{\text{ij}}+\beta_{10\,j}\,{\left(\text{PCTR}\right)}_{\text{ij}}+r_{\text{ij}}$$

(10) $$\text{Level}-2\,\mathrm{Model}:\beta_{0\,j}=\gamma_{00}+\gamma_{01}\,{\left(\text{SGCM}\right)}_{j}+\gamma_{02}\,{\left(\text{W}\,2\,\text{CACM}\right)}_{j}+\gamma_{03}\,{\left(\text{PELCM}\right)}_{j}+\gamma_{04}{\left(FES.c2CM\right)}_{j}+\gamma_{05}{\left(FES.c3CM\right)}_{j}+\gamma_{06}{\left(PP\text{CM}\right)}_{j}+\gamma_{07}\,{\left(P\,C\,T\,A\,C\,M\right)}_{j}+\gamma_{08}\,{\left(T\,C\,P\,A\,C\,M\right)}_{j}+\gamma_{09}\,{\left(P\,A\,C\,M\right)}_{j}+\gamma_{010}\,{\left(\text{PCTRCM}\right)}_{j}+\gamma_{011}\,{\left(\text{TSPR}\right)}_{j}+\gamma_{012}\,{\left(\text{NPTK}\right)}_{j}+\gamma_{013}\,{\left(\text{TPRP}\right)}_{j}+\gamma_{014}\,{\left(\text{TA}\right)}_{j}+\gamma_{015}\,{\left(\text{TG}\right)}_{j}+u_{0\,j}$$

(11) $$\beta_{\text{kj}}=\gamma_{10}+\mu_{\text{kj}}$$

Step 5: class-level predictors were added to models to examine if these class-level predictors could explain the variance of random slopes.


(12) $$\text{Level}-1\,\mathrm{Model}:Y_{\text{ij}}=\beta_{0\,j}+\beta_{1\,j}\,{\left(\text{SG}\right)}_{\text{ij}}+\beta_{2\,j}\,{\left(\text{W}\,2\,\text{CA}\right)}_{\text{ij}}+\beta_{3\,j}\,{\left(\text{PEL}\right)}_{\text{ij}}+\beta_{4\,j}{\left(\mathrm{FES}.\mathrm{C2}\right)}_{\text{ij}}+\beta_{5\,j}{\left(\mathrm{FES}.\mathrm{C3}\right)}_{\text{ij}}+\beta_{6\,j}{\left(\text{PP}\right)}_{\text{ij}}+\beta_{7\,j}{\left(\text{PCTA}\right)}_{\text{ij}}+\beta_{8\,j}\,{\left(\text{TCPA}\right)}_{\text{ij}}+\beta_{9\,j}\,{\left(\text{PA}\right)}_{\text{ij}}+\beta_{10\,j}\,{\left(\text{PCTR}\right)}_{\text{ij}}+r_{\text{ij}}$$

(13) $$\text{Level}-2\,\mathrm{Model}:\beta_{0\,j}=\gamma_{00}+\gamma_{01}\,{\left(\text{SGCM}\right)}_{j}+\gamma_{02}\,{\left(\text{W}\,2\,\text{CACM}\right)}_{j}+\gamma_{03}\,{\left(\text{PELCM}\right)}_{j}+\gamma_{04}{\left(\mathrm{FES}.\mathrm{c2CM}\right)}_{j}+\gamma_{05}{\left(\mathrm{FES}.\mathrm{c3CM}\right)}_{j}+\gamma_{06}{\left(\text{PPCM}\right)}_{j}+\gamma_{07}\,{\left(\text{PCTACM}\right)}_{j}+\gamma_{08}\,{\left(\text{TCPACM}\right)}_{j}+\gamma_{09}\,{\left(\text{PACM}\right)}_{j}+\gamma_{010}\,{\left(\text{PCTRCM}\right)}_{j}+\gamma_{011}\,{\left(\text{TSPR}\right)}_{j}+\gamma_{012}\,{\left(\text{NPTK}\right)}_{j}+\gamma_{013}\,{\left(\text{TPRP}\right)}_{j}+\gamma_{014}\,{\left(\text{TA}\right)}_{j}+\gamma_{015}\,{\left(\text{TG}\right)}_{j}+u_{0\,j}$$

(14) $$\beta_{\text{kj}}=\gamma_{k\,0}+\gamma_{111}\,{\left(\text{TSPR}\right)}_{j}+\gamma_{112}\,{\left(\text{NPTK}\right)}_{j}+\gamma_{113}\,{\left(\text{TPRP}\right)}_{j}+\gamma_{114}\,{\left(\text{TA}\right)}_{j}+\gamma_{115}\,{\left(\text{TG}\right)}_{j}+\mu_{\text{kj}}$$

Where, γ_*111*_ to γ_*115*_ represented the class-level coefficients for examining the variance in the random slopes. Only the significant coefficients would be kept in the final model.

## Results

The following results are shown based on the empirical analysis of the above survey data.

### Descriptive statistics

Table 2 shows the summary of the 8,426 students (parents), including results from both the child and class levels. Table 3 shows the descriptive statistics of the 185 classes.

**TABLE 2:** Description statistics of 8,426 child and parents.

Variables	Value	No. of cases	%
Parent-child dyad level			
SG	Female	4,006	47.54
	Male	4,420	52.46
FES	Difficult	1,538	18.25
	Medium	6,314	74.93
	Rich	543	6.44
	Missing	31	0.37
PP	Non-participation in parents’ meeting	770	9.14
	Parents’ participation/willing to participate in parents’ meeting	7,412	87.97
	Missing	244	2.90
		Mean	SD
W2-SAA	Wave 2 student academic achievement	65.30	19.26
W2-CA	Wave 2 cognitive ability	0.35	0.82
PEL	Highest parent’s education level	4.68	2.08
PCTA	Wav 1 frequency of parents contact with teachers actively in this semester	2.37	1.01
TCPA	Wave 1 frequency of teachers contact with parents actively in this semester	2.11	1.00
PA	Wave 1 extent of parents’ afraid of communicating with teachers	2.79	0.43
PCTR	Wave 1 parents cooperate with the teacher’s requirement	1.84	0.92
Class level			
SGCM	Wave 1 percentage of male students	0.52	0.07
FES.c2CM	Wave 1 percentage of difficult family	19.15	17.10
FES.c3CM	Wave 1 percentage of medium family	75.22	15.07
	Wave 1 percentage of rich family	7.48	5.44
PPCM	Wave 1 percentage of Parents’ participation/willing to participate in parents’ meeting	0.91	0.11
W2CACM	Class mean of W2CA	0.34	0.48
PELCM	Class mean of PEL	4.69	1.23
PCTACM	Class mean of PCTA	2.37	0.35
TCPACM	Class mean of TCPA	2.11	0.35
PACM	Class mean of PA	2.79	0.09
PCTRCM	Class mean of PCTR	1.65	0.28

Note. Wave 1 = 2013–2014 school year, Wave 2 = 2014–2015 school year.

**TABLE 3:** Descriptive statistics of 185 classes.

Variables		No. of cases	%
TG	Female	128	69.19
	Male	57	30.81
		Mean	SD
TA	Wave 1 teacher experiences	16.92	7.16
TSPR	Wave 1 extent of teachers’ stress from parents’ request	15.00	8.97
NPTK	Wave 1 numbers of parents that teachers know	3.58	0.87
TPRP	Wave 1 teachers’ perception of respect from parents	3.82	0.98

Note. Wave 1 = 2013–2014 school year, Wave 2 = 2014–2015 school year.

### Multilevel results

#### Results from step 1 (model 0)

From model 1 in Table 4, we can see that the mean of the students’ class academic achievements is 65.54 (*p* < 0.001), the variance of the students (σ^2^) is 513.9, and the variance of the classes is 157.9 (*p* < 0.001), thus ICC = 0.42, indicating that the application of the HLM to statistical analysis must be considered (Wen, 2009).

**TABLE 4:** Multilevel result.

	Model 0	Model 1	Model 1.1	Model 2	Model 3
Fixed effects					
Level 1-child level					
Intercept	65.540.94**	77.4722.68**	77.6222.63**	48.7123.39*	49.5523.39*
SG		–5.080.31**	–5.050.31**	–5.060.32**	–5.060.32**
CA		11.050.24**	11.020.23**	11.030.24**	11.030.24**
FES.C2		0.280.48	0.210.48	0.140.49	0.140.48
FES.C3		–0.330.73	–0.330.73	–0.460.74	–0.470.74
PEL		0.590.09**	0.570.09**	0.560.09**	0.560.09**
PCTR		–1.750.19**	–1.760.19**	–1.720.19**	–1.730.19**
PP		2.840.66**	2.820.65**	2.940.67**	2.920.67**
PCTA		0.480.18**	0.450.18*	0.410.19*	0.410.19*
TCPA		–1.830.19**	–1.770.22**	–1.780.23**	–1.950.91*
PA		3.090.37**	2.920.43**	2.990.43**	–1.821.79
Level 2-class level					
SGCM		–6.018.61	–6.728.58	–7.918.42	–7.798.42
W2CACM		14.241.8**	14.331.8**	13.311.83**	13.331.83**
FES.c2CM		0.020.07	0.030.07	–0.020.06	–0.020.06
FES.c3CM		0.280.13*	0.290.13*	0.230.13	0.230.13
PELCM		0.020.81	0.060.8	0.620.79	0.620.79
PCTRCM		–4.472.61	–4.162.61	–3.272.55	–3.32.55
PPCM		–4.878.97	–5.088.94	–1.148.93	–1.078.93
PCTACM		1.242.54	1.332.53	2.182.58	2.172.58
TCPACM		–0.042.44	0.12.44	–0.442.49	–0.412.49
PACM		–3.316.65	–3.776.64	2.426.76	2.376.76
TSPR				–1.660.74*	–1.670.74*
NPTK				0.560.7	0.410.7
TPRP				2.151.02*	2.131.02*
TA				0.120.07	0.120.07
TG				–0.131.39	–0.131.39
TCPA*NPTK					0.040.23
PA*NPTK					1.240.45**
Random effects					
level 2 intercept	157.9	53.39	53.47	49.01	48.98
level 2 slope-TCPA			2.21	2.28	2.29
level 2 slope-PA			5.89	5.98	4.65
Level 1 residual	213.9	130.18	127.06	128.10	128.10

Note. SG, student gender; FES, family economic status; PP, Wave 1 parents’ participation/willing to participate in parents’ meeting; W2-CA, Wave 2 students’ cognitive ability; PEL, Highest parent’s education level; PCTA, Wave 1 frequency of parents contact with teachers actively in this semester; TCPA, Wave 1 frequency of teachers contact with parents actively in this semester; PA, Wave 1 extent of parents’ afraid of communicating with teachers; PCTR, Wave 1 parents cooperate with the teacher’s requirement; SGCM, Wave 1 percentage of male students; FES.c2CM, Wave 1 percentage of medium family; FES.c3CM, Wave 1 percentage of rich family; PPCM, Wave 1 percentage of Parents’ participation/willing to participate in parents’ meeting; W2CACM, Class mean of W2CA; PELCM, Class mean of PEL; PCTACM, Class mean of PCTA; TCPACM, Class mean of TCPA; PACM, Class mean of PA; PCTRCM, Class mean of PCTR; TG, teacher gender; TA, Wave 1 teacher experiences; TSPR, Wave 1 extent of teachers’ stress from parents’ request; NPTK, Wave 1 numbers of parents that teachers know; and TPRP, Wave 1 teachers’ perception of respect from parents. *p < 0.05; **p < 0.01.

#### Results from step 2 (model 1)

The results of model 2 in Table 4 show that the fixed effects of the control variables on the parent level: the average score of the classes is 0.143 (*p* < 0.05) after controlling all the other variables; boys have significantly lower academic achievements than girls (γ_*01*_ = –5.08, *p* < 0.001); as expected, cognitive ability is positively related to students’ academic achievements (γ_*02*_ = 11.05, *p* < 0.001); and the parents’ education level is positively related to students’ academic achievements (γ_*03*_ = 0.59, *p* < 0.001). The effect of FES is not significant. Parents’ willingness to participate in parental meetings to discuss students’ academic achievements have significantly higher academic achievements than those showing unwillingness (γ_*06*_ = 2.84, *p* < 0.001). Students who had parents communicating more with teachers have significantly higher academic achievements (γ_*07*_ = 0.48, *p* < 0.01).

Teachers who actively communicate with parents have significantly lower academic achievements than those who do not actively communicate with parents (γ_*08*_ = –1.83, *p* < 0.001). Parents who were not afraid of communicating with teachers have significantly higher academic achievements than those who are not afraid (γ_*09*_ = 3.09, *p* < 0.001). students with parents who had less cooperation with the teachers’ requirements have significantly lower academic achievements (γ_*010*_ = –1.75, *p* < 0.05). At the classroom level, we can find that the class level cognitive ability is significantly positive, indicating that a higher level of class level of cognitive ability is associated with a higher level of academic achievement on average. Even though individual-level FES is not significantly associated with students’ achievement, the class-level percentage of rich families is significantly positively related to students’ achievement. Together, these results indicate that, in the same class, student’s FES did not impact his/her academic achievement; however, a student in a classroom with a higher percentage of rich families would have a higher academic achievement score.

The results of random effects are as follows: the class’s mean academic achievements variance was reduced to 53.39, and the individual residual variance was reduced to 130.18 after including both level-1 and level-2 predictors. Accordingly, level-1 *R*^2^ = 0.39, and level-2 *R*^2^ = 0.66, indicating that a total of 39% of the level-1 variance and 66% of level-2 variance have been explained.

#### Results from step 3 (model 1.1)

In this step, we tested a series of models to identify which level-1 coefficients varied across classrooms. As a result, we identified that TCPA and PA effects varied across classrooms. The fixed effects were kept the same as in the last model.

#### Results from step 4 (model 2)

In this step, class-level teacher predictors are included in the model to explain the variance of the class mean of students’ achievement scores. As shown in Table 4, we found that the extent to if teachers’ stress from parents’ requests is significantly negatively associated with the class mean of students’ academic scores, indicating the higher levels of stress teachers perceived, the lower average scores the class had. Also, teachers’ perception of parents’ respect significantly impacts class-level academic scores. Regarding the random effects, the variance of the class means academic achievements was reduced to 49.01 from 53.47. Accordingly, level-1 *R*^2^ = 0.40, and level-2 *R*^2^ = 0.66, indicating the effects of class-level predictors were very small.

#### Results from step 5 (model 3)

In this step, we tested if class-level predictors could significantly explain the variations of random slopes by including cross-level interactions between parent and teacher predictors. More specifically, if the number of parents that teachers know as class-level predictors could explain the effects of the frequency of teachers contact with parents actively and extent of parents’ afraid of communicating with teachers as child-level predictors (TCPA*NPTK and PA*NPTK). The results show that the numbers of parents that teachers knew are significantly positively associated with the slope of the extent of parents’ afraid of communicating with teachers, indicating that when teachers knew more parents, the impacts of the extent of parents’ afraid of communicating with teachers become more positive, as a result, parents had lower levels of afraid of communicating with teachers. Accordingly, level-1 *R*^2^ = 0.40, and level-2 *R*^2^ = 0.69, as expected, level-2 variances have been further explained by adding these cross-level interactions.

## Discussion

In this paper, we focus on the association between the parent-teacher relationship and the student’s achievements, aiming to analyze the carry-over impact of the parent-teacher relationship on students’ achievements for middle school students in China. The HLM was applied to analyze two levels of variables: the parent-child dyad level and class level. Therefore, we organize discussions about the associations of these two levels of variables, including child-level variables and class-level variables, of the parent-teacher relationship with students’ academic achievements.

### The carry-over impact of the parent-teacher relationship on students’ academic achievements at the parent-child dyad

For the parent-teacher relationship, the degree and form of parent-teacher contact are strongly associated with students’ academic achievement. Students with parents who participated in parental meetings and actively communicated with teachers had higher academic achievement, which a large number of studies have proved (e.g., Goyette and Xie, 1999; Adams and Christenson, 2000; Sebastian and Allensworth, 2012). The frequency of parent-teacher interaction could pass on educational expectations to their children, then contribute to their academic achievements.

The cooperation from parents to supervise their children to complete teachers’ assignments was an important way for parents to engage in their children’s schooling (Cook et al., 2018), which was supported by our findings as well. More specifically, results showed that the less the parents cooperated with teachers’ requirements, the lower students’ academic scores were.

Our results also showed that parental participation in parent-teacher meetings is also positively related to students’ academic achievements.

On the one hand, parental participation in parent-teacher meetings reflects the level of parent involvement in children’s learning to some extent. The positive relationship between parental involvement in their children’s education and students’ success in school is widely documented in the research literature (Fan and Chen, 2001; Barnard, 2004; Todd and Wolpin, 2007; Houtenville and Conway, 2008; Cheung and Pomerantz, 2011). On the other hand, evidence also suggests that some schools failed to fully engage parents and provide them with information about their children’s learning and how they are performing in school (Noel et al., 2013). Therefore, it is critical for parents to actively keep themselves informed about their children’s classroom activities, events, and requirements by communicating with teachers actively.

However, we found that parents reported teachers’ active communication with parents had negative relationships on students’ academic performance, which might be because, in the face-to-face communication between parents and teachers, teachers always have the clear aim of reflecting on the students’ misconduct and requesting that parents cooperate with regulations (Xiong, 2007; Zhang, 2011). If parents are informed of their children’s misconduct in China, they tend to criticize them verbally or even scold them since the children are supposed to obey the rules of parents. So that, telling parents about bad behavior is a punishment in Chinese published schools, which may aggravate students’ negative emotions, further affecting their academic performance. The relationship between teachers and parents in China is protagonist-supporting as a result of the current education system in China, which is oriented toward a screening function rather than a cultivation function (An, 2005), leading to strengthening the importance of exams and the status of teachers (An, 2005; Peterson et al., 2011). Although policymakers, administrators, and teachers have gradually realized the vital role of parental participation, parents’ involvement in children’s education is still in its initial stage in China (Wu et al., 2017).

### The carry-over impact of the parent-teacher relationship on students’ academic achievements at the class level

The results of model 3 showed that teachers’ perception of respect from parents and the extent of teachers’ stress from parents’ requests were significantly associated with academic achievement. The higher levels of stress teachers perceived from parents’ requests, the lower academic achievement; meanwhile, the higher levels of respect teachers perceived from parents, the higher average academic performance. Similar to previous meta-analysis findings, high teachers’ stress level is linked to poor student outcomes, and the parent-teacher relationship is one of the main sources of teacher stress (Cameron and André, 2005). Also, having parents’ respect is a well-known protective factor for teacher retention (Ouyang and Paprock, 2006; Canter, 2009).

In addition, results reveal the random effects of the extent of parents’ afraid of communicating with teachers could be explained by the number of parents that teacher knew; the more parents the teacher knew, the lower level of fear that parents felt about communicating with teachers would become more positive. Together, the results echo that a good parent-teacher relationship link to good academic performance of students. However, the random effects also reveal the non-negligible variation across classrooms. Teachers can teach so many students that they are not familiar with all their children and their parents (Sizer, 1992; Meier, 2005), especially when there are many large classes (more than 56 students in a class) in China (Zhou and Xian, 2012; Zhang, 2013; Fu and Xu, 2018). Hu and Luo (2014) investigated large classes in two cities in Guangxi Province and found that large classes were more abundant in middle and high schools than in primary schools; the number of students in the largest class was 103. Therefore, some classes make it difficult for teachers to be familiar with each student’s parents in China. Yang (2006) found 45.2% of parents contacted teachers when their children fell behind, 19.4% of whom contacted the teacher when their children exhibited problem behavior, while 13.7% took the initiative to communicate with teachers only when the children had special conditions (such as sickness, incomprehension of fees, opinions on teaching methods, and corporal punishment). Thus, the current results also highlight the inequity of education resources, especially the human resources of teachers, in middle schools.

## Practical implication

We found the parent-teacher relationship has a carry-over impact on middle school students’ academic achievements both at the parent-child dyad level and at the class level. It’s important to take measures to promote the parent-teacher relationship for middle school students. First, teachers should develop an equal dialogue with parents and guide parents to take the initiative to participate in school education. It’s critical to express welcome and affirmation for parents’ involvement and avoid parents having fear of communication with teachers. Second, parents should expand the communication channels with teachers, including the participation in school activities, volunteer activities, and so on. Third, the schools should provide a creative, open, inclusive atmosphere to attract parents to participate in the children’s education and timely communication with the school about the student’s important performance. Promote the healthy interaction between parents and teachers to form a habit and even become a part of the school culture.

## Limitations and future research directions

Some limitations of this research should be noted. First, the results concerning ethnicity should be interpreted with caution, as all minorities were treated as one group. Second, the data only covered students in seventh grade, and the influence of the parent-teacher relationship may be different for students in other grades (Fan, 2021). With the increase of grade, children’s autonomy gradually improves, and their openness to parents’ opinions gradually decreases, resulting in a decline in the influence of parental involvement on middle school children (Gao, 2016).

To address these issues, future research should attempt to compare the differences in the influence of parent-teacher relationships in different grades. Moreover, with the emphasis on students’ comprehensive quality development in China, students’ development is not limited to academic success but also career and social development. Thus, future studies are needed to examine the impact of the parent-teacher partnership on other cognitive, behavioral, and social outcomes as well as the linking mechanisms among Chinese students.

## Data availability statement

The original contributions presented in the study are included in the article/supplementary material, further inquiries can be directed to the corresponding author.

## Ethics statement

Ethical review and approval was not required for the study on human participants in accordance with the local legislation and institutional requirements. Written informed consent from the patients/participants was not required to participate in this study in accordance with the national legislation and the institutional requirements.

## Author contributions

WF conceived the study, conducted the analysis, took the lead on drafting the manuscript and revised it critically for important intellectual content. QP analyzed the data and revised it critically for important intellectual content. YY contributed to the design of the work and wrote the draft of the manuscript. GC interpreted data for the work and wrote the draft of the manuscript. All authors gave final approval of the version to be published and agree to be accountable of all aspects of the work in ensuring that questions related to the accuracy or integrity of any part of the work are appropriately investigated and resolved.

## Footnotes

1 Parent-child dyad refers to the pairing of a child with his/her parents.
